# Genotypic characterization of HIV variants from PBMCs and spermatozoa

**DOI:** 10.1186/1471-2334-14-S3-E9

**Published:** 2014-05-27

**Authors:** Jyoti P Sutar, Varsha S Padwal, Shilpa M Velhal, Atmaram H Bandivdekar

**Affiliations:** 1 Department of Biochemistry, National Institute for Research in Reproductive Health, Mumbai, India

## Background

Several studies have shown that detectable levels of HIV are observed in semen in spite of undetectable viral load in blood following antiretroviral therapy (ART). Also, different HIV variants have been detected in blood and semen of the same individual, suggesting that drugs may not be uniformly effective to control the viral load and infectivity in different tissues and secretions, which in turn may affect sexual transmission of HIV. Hence, genotypic characterization of HIV variants in PBMCs and sperm of the same individual may provide information about the association of these variants with sexual transmission of HIV.

## Methods

Present study describes characterization of translated amino acid sequence of C2 v3 region of *env* of HIV-1 C in PBMCs and Spermatozoa of 14 and 5 individuals, respectively. The sequences were analysed for presence of N linked glycosylation sites using the program N-Glycosite. Also, Co receptor usage predictions were done using an online tool, Geno2pheno [Co receptor] 1.2.

## Results

Significant differences in the number of N linked glycosylation (NLG) sites were observed in PBMCs and spermatozoa of the same individuals as well as samples collected from different individuals.

## Conclusion

This study describes presence of compartmentalization between blood and semen which may influence the effect of ART in control of HIV/AIDS and influence sexual transmission of HIV.

